# Breaking barriers in male infertility: the power of artificial intelligence-driven solutions

**DOI:** 10.3389/fruro.2026.1719894

**Published:** 2026-03-20

**Authors:** Laura Ibañez Vazquez, Natalia Pérez Romero, Daniel Tueti Silva, Sarelis Infante, Claudia González-Santander, Irene De La Parra, Isabel Galante Romo, Juan A Gómez Rivas, Jesús Moreno Sierra

**Affiliations:** 1 San Carlos University Clinical Hospital, Madrid, Spain; 2 Hospital de Nuestra Señora de Sonsoles, Avila, Spain; 3 Hospital Universitario Ramon y Cajal, Madrid, Spain

**Keywords:** artificial intelligence, male infertility, predictive models of art, semen analyses, semen diagnostic evaluation

## Abstract

Infertility is defined as the inability of a sexually active couple, not using contraception, to achieve a spontaneous pregnancy within 12 months. It affects an estimated 8% to 12% of couples worldwide, with 30% to 50% of cases attributable, either primarily or in part, to male factors. Despite the increasing number of assisted reproductive technology (ART) procedures performed globally, improvements in fertilization and pregnancy outcomes have been limited. The need to improve diagnostic accuracy and therapeutic efficiency has driven the development of artificial intelligence (AI) in reproductive medicine. This narrative review aims to explore how AI is transforming the diagnosis and treatment of male infertility. AI technologies are nowadays being used to automate and refine semen analysis, providing more reliable assessments of sperm morphology, motility, and concentration. These innovations enable clinicians to improve the prediction of semen quality and to identify which patients might benefit most from specific interventions, such as sperm retrieval in cases of non-obstructive azoospermia or the selection of optimal sperm cells for reproductive techniques. Moreover, advanced AI algorithms—including support vector machines, deep neural networks, and decision trees—outperform traditional methods, offering greater precision and reducing subjectivity in laboratory evaluations. Additionally, AI is being utilized to estimate the chances of success with assisted reproductive techniques, assess sperm DNA fragmentation, and guide the selection of sperm. The integration of AI into clinical practice not only enables more accessible and personalized diagnoses but also opens new perspectives for the development of individualized treatments, optimizing reproductive outcomes. However, further multicenter validation of AI-based models, methodological standardization, and careful consideration of ethical and privacy issues are necessary before widespread clinical adoption.

## Introduction

The World Health Organization (WHO) recently reported that approximately one in six people will experience infertility at some point during their reproductive years. This amounts to approximately 17% of the global adult population. Although prevalence varies across regions and sociodemographic levels, the global burden continues to rise (1).

Infertility is defined as the failure to establish a clinical pregnancy after 12 months of regular, unprotected sexual intercourse, or as an impairment in a person’s capacity to reproduce, either individually or with a partner (2). Note that subfertility can be used interchangeably with infertility (2), whereas sterility refers to permanent infertility (2).

Infertility can have a female or male origin. However, among all diagnosed infertility cases, approximately 30% are due to male infertility, with a prevalence of 9% to 15% in the general male population according to epidemiological studies (3). Consequently, male infertility is increasingly recognized as a primary etiological component of reproductive dysfunction. Research into discovering mechanisms and potential therapeutic approaches is intensifying.

Male infertility is associated with a range of congenital abnormalities, genetic disorders, environmental exposures, and lifestyle-related risk factors. Poor dietary habits, varicocele, systemic diseases, or malignancies such as testicular cancer, ejaculatory duct obstructions, and viral or bacterial infections have all been shown to negatively impact sperm quality, including infections caused by the human papillomavirus (HPV), SARS-CoV-2, and other urogenital pathogens, which have all been associated with reduced sperm parameters. This can result in increased deoxyribonucleic acid (DNA) fragmentation and suboptimal outcomes in assisted reproductive techniques (ARTs) (4, 5). However, few studies to date have systematically aggregated and prioritized the potential causes and risk factors of male infertility. These developments have promoted the establishment of a consensus on its causal pathways and supported the development of straightforward preventive and therapeutic strategies (6). Due to the often asymptomatic nature of male infertility, diagnosis is mainly based on semen analysis. Epidemiological evidence has reported a progressive decline in sperm counts over the past several decades (7), underscoring the increasing contribution of male factors to the global burden of infertility (8). Numerous studies have established that abnormalities in sperm parameters, such as low sperm concentration (azoospermia or oligozoospermia), reduced motility (asthenozoospermia), and atypical morphology (teratozoospermia), represent major etiological factors in male infertility worldwide (9).

ARTs such as intracytoplasmic sperm injection (ICSI) and *in vitro* fertilization (IVF) are generally recommended when semen parameters are severely compromised. Since the first IVF-assisted births in 1978 (10) and the first ICSI procedure in 1992 (11), new ART techniques have been continuously developed and implemented in clinical practice. In addition, ongoing modifications and refinements to existing techniques have been developed. These innovations seek to improve fertilization outcomes and address the complexity of underlying reproductive issues, offering more tailored solutions for patients. The dynamic evolution of ART reflects the growing understanding of reproductive biology and the constant pursuit of more effective and individualized fertility treatments. Fertilization rates with standard IVF significantly decline when sperm concentration is below 5 million/mL, especially when normal morphology is below 1%, making ICSI the preferred approach. Although ICSI has a relatively high success rate (with up to 60% of women achieving birth), it is a more invasive procedure than traditional IVF. One risk is that approximately 2% of eggs may be damaged during the sperm injection process, making them non-viable. Preparing for ICSI also requires greater precision: embryologists must carefully select a single healthy sperm under a microscope and inject it into the egg using a fine needle (12). Due to its complexity, ICSI has revolutionized the treatment of severe male factor infertility by enabling fertilization with even a single viable spermatozoon, thereby overcoming critical barriers related to low sperm count, poor motility, and abnormal morphology (4). Its clinical use has expanded from epididymal sperm to testicular sperm retrieved via biopsy, even when the sperm are immotile or in immature forms like spermatids. Remarkably, ICSI has also allowed fertilization with round-headed or acrosome-lacking sperm, which are otherwise incapable of penetrating the oocyte (13). While there is some consensus, universal criteria for patient selection in assisted reproduction remain undefined. In the pursuit of improved ART outcomes, selecting high-quality spermatozoa represents a critical step. Effective sperm selection techniques are designed to identify and isolate sperm cells that exhibit optimal motility and normal morphology and have intact genetic material. This is because these parameters are closely associated with a higher fertilization rate and better embryonic development.

The overarching goal is to enhance both the efficiency of fertilization and the likelihood of achieving viable, high-quality embryos suitable for transfer. As a result, conventional semen analysis continues to serve as the cornerstone of initial male fertility assessment. This includes evaluating sperm concentration, motility, and vitality, as well as morphological examination, which provides essential diagnostic information regarding male reproductive potential. Subsequently, healthy sperm can be selected using methods like hyaluronan binding, centrifugation, magnetic separation, or even simple, compact devices. While these approaches are generally sufficient for IVF, the success rates are lower, with only approximately 30%–35% of women achieving pregnancy (12). Consequently, there is a growing interest in the functional and molecular quality of sperm, leading to the development of more advanced selection techniques. These include density gradient centrifugation, swim-up methods, and more recently, microfluidics and magnetic-activated cell sorting (MACS). These techniques aim to further refine the isolation of sperm with the highest reproductive competence (4).

**Figure 1:**
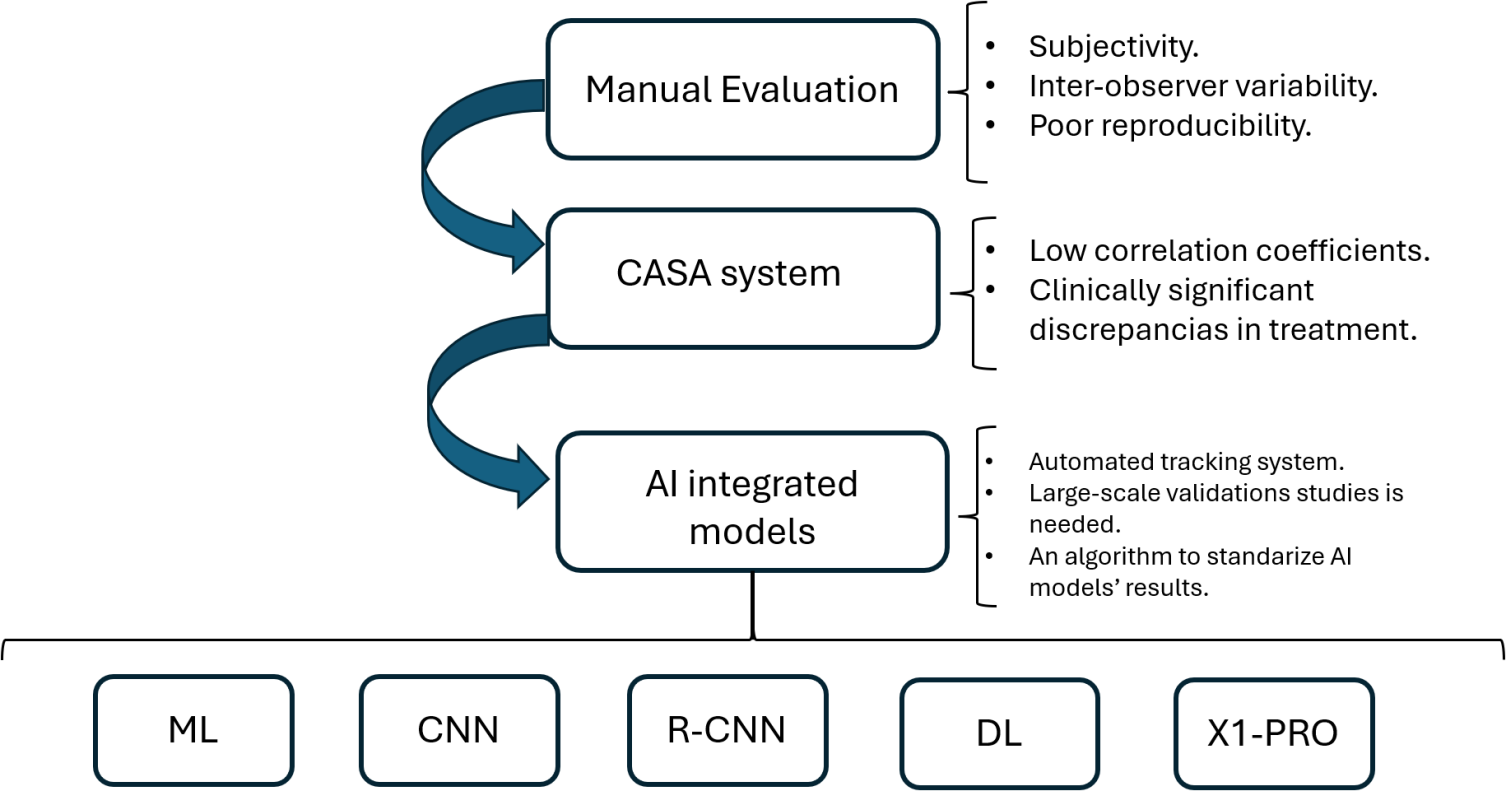
Technology development in male infertility. The evolution of AI technologies in male infertility and their limitations at each stage.

For these reasons, artificial intelligence (AI) is rapidly transforming the landscape of reproductive medicine, particularly regarding the diagnosis and management of male infertility. AI-driven tools offer a novel approach to sperm evaluation and selection by addressing the inherent limitations of traditional assessments, such as subjectivity, interobserver variability, and limited analytical depth (14). In this context, AI has demonstrated strong potential in automating sperm analysis by accurately assessing key parameters such as morphology, motility, and DNA integrity. This reduces reliance on manual microscopy and minimizes human error and subjectivity. Among other procedures, AI leverages machine learning (ML), artificial neural networks (ANNs), and deep learning (DL), which can process and interpret large and complex datasets derived from clinical records, imaging, and genetic profiles. ML uses data-driven algorithms to distinguish and interpret complex sequences. Rules are not exactly coded, and ML relies on training data to identify the most suitable statistical model for a given task. Four types of ML can be distinguished: supervised, unsupervised, semisupervised, and reinforcement learning. ANN is a subfield of ML. It includes input nodes, output nodes, and several hidden layers. These nodes act as nerve cell bodies, communicating with each other by connections that are analogous to axons or dendrites. The correlated outputs and connections between nodes are weighted to strengthen the relation. Meanwhile, DL networks are advanced artificial neural networks with multiple nodes that reveal hidden and abstract relationships. The new output is processed alongside the previous one to extract more complex associations. However, these models are more difficult to interpret, generating the “black box problem” (clear input with unclear output) (15) (**Figure 1**).

Unlike conventional statistical models, AI algorithms can identify non-linear relationships and subtle patterns that may be imperceptible to the human eye. This enhances diagnostic precision and clinical decision-making (15). Furthermore, AI-assisted image analysis systems can efficiently screen sperm and embryos, providing consistent and reproducible grading. Additionally, AI algorithms can also integrate clinical data with laboratory outcomes to predict the likelihood of successful fertilization, blastocyst development, and implantation. This contributes to more personalized and effective treatment strategies (16). These innovations are particularly valuable in challenging cases, where AI can assist in identifying viable sperm within testicular biopsies. Additionally, predictive modeling using AI can support treatment planning by identifying patients who are most likely to benefit from specific interventions, such as varicocele repair or hormonal therapy. This helps avoid unnecessary procedures and optimize resource allocation (16).

## Aims of the review

Although the clinical application of AI in male infertility is still emerging, this review aims to critically examine and synthesize the findings of current studies on its diagnostic and therapeutic potential. It seeks to contextualize male infertility within its broader epidemiological and clinical landscape, highlighting the increasing contribution of male factors to the global burden of infertility and the limitations of conventional diagnostic and sperm-selection methods. The review explores how AI-based tools may overcome these limitations by improving the accuracy, consistency, and depth of sperm evaluation and by supporting more precise predictions of assisted reproductive outcomes. In addition, it examines the main AI methodologies currently applied in reproductive medicine, outlining their advantages, constraints, and interpretability challenges. Furthermore, this review aims to identify emerging applications such as algorithm-guided treatment planning or automated image-analysis systems and to discuss their potential to enhance personalized fertility care. Finally, the review considers current gaps, ethical considerations, and priority areas for future research to support the responsible and effective implementation of AI in the management of male infertility.

## Methodology

A narrative literature review was conducted using a non-systematic, integrative approach to explore the current landscape of AI applications in the diagnosis and management of male infertility. This design was chosen to comprehensively summarize the emerging and diverse body of literature in this field, encompassing both clinical and technical studies. A broad but focused search strategy was adopted to identify relevant peer-reviewed articles, clinical studies, reviews, and technological developments addressing the intersection between AI technologies and male reproductive health.

### Literature search

A comprehensive search was performed in major scientific databases including PubMed, Scopus, Web of Science, and Google Scholar. The search covered literature published between January 2010 and June 2025, with a particular focus on more recent literature from the last 5 years. The search was last updated on 15 August 2025.

The primary keywords included combinations of “male infertility,” “artificial intelligence,” “machine learning,” “deep learning,” “ART,” “sperm selection,” “andrology,” and “urology.” Boolean operators were used to refine and expand the search strategy as appropriate. After the initial search, more than a thousand records were identified (PubMed: 319; Scopus: 188; Web of Science: 209; Google Scholar: 355). Following title and abstract screening, 53 studies were included in the final analysis.

### Inclusion and selection criteria

Articles were selected based on their relevance to the core themes of the review: the role of AI in semen analysis and sperm selection, predictive models of ART outcomes, and clinical decision support systems in andrology and reproductive medicine. This review not only focuses on the clinical and technological applications of AI but also evaluates its advantages, limitations, and future perspectives in male reproductive health.

Due to the narrative nature of this review, no formal quality assessment tools such as Preferred Reporting Items for Systematic Review and Meta-Analysis (PRISMA) or Grading of Recommendations Assessment, Development, and Evaluation (GRADE) were applied. Instead, the selection process was guided by clinical relevance, scientific rigor, and contribution to the overall understanding of AI in male infertility. Publications providing either original data or significant conceptual contributions, such as recent meta-analyses, consensus statements, and high-quality reviews, were prioritized. Only articles published in English and accessible in full text were included. Non-peer-reviewed materials, opinion pieces, and studies without sufficient methodological transparency were excluded.

### Data organization and thematic analysis

The literature included was analyzed thematically and organized into several key domains:

1. AI in semen analysis and diagnostic evaluation.
2. Predictive models of ART outcomes.
3. The advantages of AI in the management of male infertility.
4. The disadvantages of AI in the management of male infertility.

This review does not aim to provide an exhaustive list of all available publications, but rather to critically synthesize relevant and high-impact knowledge from a diverse body of research. The objective is to critically evaluate the current evidence and highlight the role of artificial intelligence in diagnosing and managing male infertility, as well as its future directions.

## AI in semen analysis and diagnostic evaluation

Semen analysis plays a key role in evaluating infertility (17). According to WHO guidelines, a standard semen analysis should assess several factors including semen volume, sperm concentration, total sperm count, motility, morphology, and vitality (18). However, traditional semen analysis relies heavily on manual evaluation, which introduces subjectivity and variability, resulting in poor reproducibility. This makes it difficult to accurately evaluate crucial sperm parameters such as morphology, motility, and concentration, all of which are essential for accurate diagnosis and the successful planning of treatment strategies.

AI has the potential to improve diagnostic accuracy by automating sperm evaluation, reducing variability, and identifying abnormal sperm characteristics more consistently than manual methods. In addition, AI-based predictive models can integrate a wide range of data including clinical information, imaging, and patient history to more accurately predict outcomes such as sperm retrieval success, fertilization potential, and IVF results (14). It is also promising that these tools are beginning to consider multiple types of information like clinical records, medical images, videos, and tubular waveform data, in order to provide a more comprehensive analysis of fertility potential. Various ML techniques have been applied to sperm image analysis in infertile patients, including multilayer perceptron (MLP), support vector machines (SVMs), logistic regression, random forest, gradient boosting trees, least absolute shrinkage and selection operator (LASSO), extreme gradient boosting (XGBoost), and different types of neural networks. These models have been used to study sperm-related factors like head morphology, motility, pH, and overall quality (16).

Nonetheless, a common issue is that most automated methods for evaluating sperm motility and morphology tend to focus on just one aspect at a time, without considering other relevant data. Moreover, these evaluations often rely on limited or isolated datasets, making it challenging to reproduce or compare the results across different studies (17).

### Morphological analysis using AI

Sperm morphology is usually assessed by examining samples for abnormalities in the head, neck, midpiece, and tail, as well as by identifying any excess residual cytoplasm (17). The WHO guidelines provide reference ranges for several semen parameters, based on samples collected from fertile men (18). Manual semen analysis must be performed by trained laboratory personnel, but even when conducted in accordance with WHO standards, considerable variation can still be observed both within and between laboratories. Furthermore, some investigators argue that morphological characteristics by themselves are not a reliable indicator for distinguishing fertile men from infertile men (17). Moreover, sperm morphology alone has not been shown to directly impact ART outcomes (19).

However, efforts to automate semen analysis in order to improve results have been ongoing for decades. In 1980, computer-assisted sperm analysis (CASA) emerged with advances in imaging digitalization (17). These systems are designed to provide objective, quantitative assessments of various aspects of sperm structure and function, with the goal of improving consistency within and between laboratories (20). However, achieving consistent and accurate results has proven challenging (21). CASA systems have been expanded to include evaluations of sperm morphology and DNA fragmentation, with more recent versions claiming to offer insight into sperm vitality and enable certain functional assessments. However, they still require specialized staining techniques or additional sample preparation, which limits their practicality. Additionally, the main challenges are interference from non-sperm cells or particles in the sample and complications caused by sperm collisions or overlap during movement analysis (17). Despite its long-standing use, CASA has not been widely adopted in clinical practice (17). There is poor agreement with manual methods for morphology assessment, with low correlation coefficients and clinically significant discrepancies in treatment allocation (12, 22). But CASA technology continues to advance. One practical advantage is its ability to generate a wide range of sample images reflecting different sperm characteristics. These images can be used for teaching and training purposes, eliminating the need to handle real semen samples (20).

Due to all these factors, many AI models have been developed. Some have focused specifically on analyzing the morphology of sperm heads (23–25), while others have attempted to recognize structural features across the entire sperm cell (26, 27). These differences in focus and methodology make it challenging to compare the results directly or draw clear conclusions about their clinical relevance. Also, the datasets used are often quite small, including only a limited number of sperm cells or patient samples (19). The development of several ML techniques, such as MLP, SVM, and DL models, has been applied to sperm images from infertile patients, with the aim of classifying sperm head morphology based on shape and structural features (16, 28, 29). These models can process fresh human sperm in real time at magnifications ranging from ×400 to ×600 (19). Moreover, Hicks et al. argued that DL methods could be used as an effective support tool in human semen analysis (17). Compared to traditional manual methods, ML and DL models have demonstrated improved performance in analyzing key sperm parameters. Notably, the SVM model reached an area under the curve (AUC) of 88.59% and a precision rate of 90% in classifying sperm morphology, positioning it as a valuable tool for infertility diagnosis (16).

Although AI models have the potential to support semen analysis, their current limitations must be recognized. As previously mentioned, the wide variation in study approaches and methodologies makes it hard to compare results or assess their utility in a clinical setting. Moreover, many of the datasets used are quite small, often involving only a limited number of sperm cells or patient samples. Because of all of this, the routine use of these methods for evaluating sperm morphology in current clinical practice is not recommended.

A summary of the evidence in this domain is provided in **Table 1**.

**Table 1:** A summary of evidence on morphological analysis using AI.

Article	Study design	Number of centers	Sample size	Validation level	Evidence level
K Qaderi et al. (16)	Narrative review of mapping	–	14 studies included	–	Low
SA Hicks et al. (17)	Retrospective study of ML	Single center (OsloMet)	85 semen videos	Internal; no external validation	Low
MA Riegler et al. (19)	Narrative review of AI in ART	–	–	–	Low
JW Choi et al. (20)	Modeling and simulation study	Single center (NJIT/Drexel)	Synthetic simulations + small validation set	Internal; no external validation	Low
ST Mortimer et al. (21)	Narrative review of CASA	–	–	–	Low
M Xu et al. (22)	Prospective clinical diagnostic accuracy study	Single center (University of Hong Kong)	CASA images from 326 semen samples (326 patients)	Internal; no external validation	Low–moderate
V Chang et al. (23)	Retrospective study of ML	Single center (Chile SCIAN-Lab)	SCIAN dataset (1,200 images)	Internal; no external validation	Low
F Shaker et al. (24)	Retrospective study	Single-center collection (IFIC)	HuSHeM dataset (216 head images), SCIAN dataset (1,133 images)	Internal; no external validation	Low–moderate
J Riordon et al. (25)	Retrospective study of DL classification	Single center (University of Toronto)	SCIAN (1,200 images), HuSHeM (1,600 images)	Internal; no external validation	Low–moderate
RA Movahed et al. (26)	Retrospective study of image-based segmentation study	Single center (University of Tarbiat Modares)	HuSHeM (216)	Internal only; no external dataset	Low
HO Ilhan et al. (27)	Retrospective study of ML with advanced preprocessing	Single center (University of Pamukkale)	HuSHeM (216), SMIDS (3,000)	Internal; no external validation	Low–moderate
S Javadi et al. (28)	Retrospective study of the development and testing of AI	Single center (University of Guilan)	MHSMA dataset (1,540 images/235 patients)	Internal; no external validation	Moderate
A Abbasi et al. (29)	Retrospective computational modeling study	Single center (University of Guilan)	MHSMA dataset (1,540 images)	Internal; no external validation	Low–moderate

### Sperm DNA integrity

DNA integrity is a significant qualitative measure of sperm quality. It has been reported to be a determinant of reproductive outcomes (30).

DNA fragmentation, telomere shortening, and chromosome abnormalities often damage the DNA in the head of the sperm. Sperm genetic analysis is an important part of diagnosing male factor infertility. Identifying the factors that cause DNA damage could be the key to improving reproductive health and developing personalized therapies (14).

Several lifestyle and environmental factors can create conditions that can damage DNA integrity and decrease the quality of sperm. Fertility is inversely correlated with the DNA fragmentation index (DFI). These conditions can lead to embryo arrest, implantation failure, miscarriage, and congenital malformations (14).

The current assays available for DNA fragmentation are invasive and preclude the use of a single sperm for ART such as the sperm chromatin structure assay, chromatin dispersion tests, and single-cell gel electrophoresis (14, 30). These procedures are highly subjective and time-consuming. Consequently, researchers have tried to automate the identification of sperm cells based on DNA integrity using a non-invasive, reliable method (15). AI algorithms could potentially correlate sperm DNA integrity with morphology in a non-invasive way (30).

The first evidence that sperm morphology could predict the DNA content of a single sperm was provided by Wang et al. (31) ML algorithms were created based on their data and images of sperm to predict DNA fragmentation (14).

McCallum et al. introduced a DL convolutional neural network (CNN) trained on sperm images linked to corresponding DFI values. Their model demonstrated a statistically significant correlation between the predicted and laboratory-measured DFI (14, 15); however, it was trained on relatively small, specific datasets and showed variable performance across morphological subgroups. While this study represents an important proof of concept, current image-based DFI prediction should be considered an adjunctive tool rather than a replacement for biochemical assays. Large-scale, multicenter studies that integrate clinical outcomes such as fertilization rate, miscarriage, and live birth are still required before this approach can be validated for clinical application.

Another limitation is the current inability to analyze subcellular semen parameters, such as the acrosome and vacuoles. These parameters play an important role in sperm quality and reproductive outcomes. Integrating data on these subcellular morphological features with standard semen parameters and DNA fragmentation measures could be a powerful way of identifying the most suitable sperm for ICSI (15).

Similarly, such systems could be used in smartphones to automate and standardize the identification of sperm DNA fragmentation and morphology, even in settings with limited resources. However, future research requires more extensive datasets, including DFI measurements (14).

A summary of the evidence in this domain is provided in **Table 2**.

**Table 2:** A summary of evidence on sperm DNA integrity.

Article	Study design	Number of centers	Sample size	Validation level	Evidence level
P Cherouveim et al. (14)	Systematic review of sperm DNA fragmentation testing and ART outcomes	–	101 studies included	–	Moderate
J Marinaro et al. (15)	Retrospective study of AI model development (CNN predicting DFI from images)	Single center (University of British Columbia)	583 sperm images (169 samples)	Internal only; no external dataset	Low–moderate
P Diaz et al. (30)	Prospective observational clinical study	Single center (Virgen de las Nieves Hospital)	120 men	–	Low
Y Wang et al. (31)	Retrospective study of ML predicting DNA fragmentation using sperm morphology	Single center (Fuqing City Hospital)	300 semen samples	Internal only; no external dataset	Low–moderate

### Sperm motility assessment

While the relationship between sperm morphology and outcomes after ART remains less clearly defined, sperm concentration and motility are typically the primary parameters considered when determining whether to use IVF or ICSI for the fertilization method (32).

Although CASA systems are widely recognized for producing consistent and reliable measurements of sperm concentration and motility, their accuracy in evaluating motility and morphology as previously mentioned remains problematic. Studies have shown only weak correlations between CASA-based motility assessments and those obtained through manual examination. In many cases, it results in different clinical classifications, which can potentially alter treatment decisions and patient management. This limitation is one of the reasons why alternative methods and more advanced techniques are being investigated (12, 22).

In the field of automated semen analysis, Urbano et al. developed a fully automated tracking system capable of following hundreds of sperm cells at the same time (33). Beyond simple tracking, the system can monitor motility parameters over time with minimal operator involvement. This is achieved through a customized version of the joint probabilistic data association filter (JPDAF) that has been adapted to work with microscopic recordings of semen. This modification enables the algorithm to distinguish and follow individual spermatozoa, even when they are close together or collide, situations which often challenge traditional CASA tools. While this represents a significant technical advance, the validation of this method on only two semen samples raises questions about how well it would perform across a broader population (17).

However, given that good sperm motility is critical to the success of ART, a computer-aided method capable of determining sample suitability from live microscope imaging can be highly valuable. Recent advances in AI, such as CNN and other DL techniques, have already shown strong potential in biomedical image analysis. This supports applications ranging from disease classification and edge detection to image segmentation, knowledge inference, image reconstruction, and shape recognition (12).

A related approach was presented by Dewan et al., who also used microscopic video to generate sperm cell trajectories (34). In their system, grayscale edge detection is first applied to identify movement paths. These trajectories are then classified by a CNN as either “sperm” or “non-sperm.” For those identified as sperm, the system estimates three motility categories: progressive, non-progressive, and immotile, as well as sperm concentration per unit volume. The reported results are promising. However, since the evaluation relied on a closed dataset, it is difficult to make direct comparisons with other existing techniques (17, 34). Additionally, Hicks et al. examined in their work CNNs to analyze video sequences of semen samples under a microscope. These models aimed to classify sperm motility into the aforementioned categories. Video data were combined with participant-related information to explore whether a multimodal approach could improve prediction accuracy (17). Thambawita et al. proposed a two-stage framework in which an autoencoder was first employed to extract key features from sperm images, followed by a pretrained ResNet34 CNN to predict both motility and head morphology (35). Despite their promise, these models are not yet entirely autonomous, as they still rely on manually annotated training images, which is both time-consuming and labor-intensive (12, 36).

A new DL-based approach has been designed to evaluate whether a semen sample meets the motility criteria for use in artificial insemination, based on microscopic video footage. This method combines a faster region-based convolutional neural network (R-CNN) for precise sperm head detection and segmentation with a heuristic algorithm to assess motility. In this context, good motility is defined as rapid and consistent movement of sperm heads in a single direction, whereas poor motility is characterized by slow or erratic movement. The R-CNN-based detection system was evaluated using a sperm video dataset, achieving an average difference of only 2.92% compared to experimental motility measurements with an accuracy of 91.77% for sperm head detection. Nonetheless, the method occasionally failed to recognize sperm heads, often due to occlusion or the presence of similar-looking artefacts, or when the heads were positioned against the frame border. One likely contributing factor was that the evaluation was conducted on relatively short video fragments because full-length videos could not be used due to shifts in the imaging area during recording. This situation caused changes in the tracked objects that the algorithm did not register (12).

Alongside sperm concentration, the development of a novel automated artificial intelligence optical microscopic (AIOM)-based technology, LensHooke™ X1 PRO (X1 PRO), showed better results in detecting sperm motility than manual evaluation. After analyzing 135 cases, the Spearman correlation was found to be *r* = 0.93. However, the study revealed that the analyzer tended to overestimate total motility slightly (37). On the other hand, for progressive motility, the regression analysis of 100 cases presented a more moderate Spearman correlation of *r* = 0.81 for progressive motility. Overall, these results indicate that the X1 PRO provides highly comparable total motility measurements to the manual method, while some discrepancies remain for progressive motility. These results highlight an area where further refinement could be beneficial (37).

In conclusion, the limitations of CASA systems in evaluating motility and morphology have restricted their clinical impact. However, recent progress in automated tracking systems has shown encouraging results. Algorithms such as CNN and R-CNN models, as well as multimodal frameworks, have demonstrated the ability to detect, classify, and quantify sperm motility with a higher level of accuracy than manual methods. These tools also provide opportunities for analyzing complex movement patterns that traditional systems often fail to capture. However, many current methods rely on small datasets, so their performance in real-world conditions remains uncertain. To enhance reproducibility and comparability, future studies should explicitly report minimum video acquisition parameters, including frame rate, magnification, video duration, temperature control, and camera settings. Authors are also encouraged to use and cite publicly available multimodal datasets such as VISEM as benchmarking resources to facilitate cross-study validation and transparency. To support standardization, a brief reporting checklist could be incorporated into future publications of motility-AI studies to ensure that these acquisition parameters are consistently documented. Integrating AI into the study of male fertility offers a promising path with more objective and reproducible measurements. Moreover, the value of semen analysis in ART outcomes could be better predicted. To achieve these objectives, larger-scale validation studies and the development of standardized algorithms and reporting frameworks will be required.

A summary of the evidence in this domain is provided in **Table 3**.

**Table 3:** A summary of evidence on sperm motility assessment.

Article	Study design	Number of centers	Sample size	Validation level	Evidence level
V Valiuškaitė et al. (12)	Retrospective study using faster R-CNN	Single center (University of Kaunas)	VISEM dataset (85 video samples)	Internal only; no external dataset	Moderate
SA Hicks et al. (17)	Retrospective DL study using CNNs for motility classification	Single center (University of Oslo Metropolitan)	VISEM dataset (85 video samples)	Internal only; no external dataset	Moderate
M Xu et al. (22)	Prospective study of diagnostic accuracy comparing the automated system and manual CASA	Single center (The Chinese University of Hong Kong)	326 semen samples	External comparison	Moderate
LF Urbano et al. (33)	Algorithm-development study of automated sperm tracking	Single center (University of Drexel)	2 human semen samples; 717 sperm cells	Internal only; no external dataset	Low
K Dewan et al. (34)	Retrospective study of AI in sperm motility	Single center (clinical lab, India)	100 semen samples	Internal only; no external dataset	Low–moderate
TB Haugen et al. (35)	Retrospective study of DL using autoencoder and CNN to predict sperm motility and morphology from video	Single center (SimulaMet, Metropolitan University of Oslo)	VISEM dataset (85 video samples)	Internal only; no external dataset	Moderate
A Agarwal et al. (37)	Prospective study of diagnostic accuracy evaluating LensHooke™ X1 PRO device and manual method	Multicenter (4 hospitals of Taiwan)	135 semen samples	External comparison	Moderate–high

## Predictive models of ART outcomes

To predict the results of male infertility treatments, different AI models have been applied, including ANN, SVM, random forest, XGBoost, DL, and Bayesian networks. ANNs incorporate variables such as age, semen parameters, and embryo quality to predict pregnancy and live birth rates in IVF/ICSI techniques (38). SVM has been used in recovery prediction and spermatic retrieval success in non-obstructive azoospermia techniques such as microsurgical testicular sperm extraction (mTESE). The advantage of random forest and XGBoost is that they detect non-linear interactions between factors. They analyze multiple clinical, hormonal, and genetic variables and predict the probability of success in intrauterine insemination (IUI) and fertilization rate in ICSI. Additionally, DL convolutional models have been applied to more objective embryo selection (16).

### Predictive models to assess the probability of success in IVF treatments

There are models used to assess the probability of success in IVF treatments. They focus primarily on estimating the probability of live birth, both for each individual cycle and cumulatively over several cycles. Available scientific evidence shows that AI-based predictive models can outperform traditional estimation methods in accuracy by integrating large volumes of clinical, biological, and even imaging data.

Studies combine clinical, morphological, and imaging data, using techniques such as DL and SVM, to generate more accurate predictions of clinical pregnancy or live birth.

The most used algorithms include random forest, XGBoost, LightGBM, deep neural networks, and logistic regression models. These models integrate key clinical variables such as maternal age, ovarian reserve [anti-Müllerian hormone (AMH), antral follicle count], sperm parameters, duration of infertility, stimulation protocols, and morphological characteristics of embryos, among others (39).

The accuracy of these models varies depending on the type of data and the approach. For example, the forecasting outcomes of assisted reproductive treatments using the artificial neural network (FORTUNE) model, based on neural networks and validated in independent cohorts, classifies patients into prognostic groups based on the probability of obtaining euploid blastocysts, with AUCs greater than 0.83 for the prediction of ≥1, ≥2, and ≥3 euploid blastocysts (40). Other models, such as XGBoost and light gradient-boosting machine (LightGBM), have reported AUCs as high as 0.999 for pregnancy and 0.913 for live birth in clinical cohorts (41).

Integrating embryo images with clinical data using fusion models has been shown to improve predictive capacity compared to models based only on clinical data or images (42).

The Life Whisperer model showed a sensitivity of 70.1% for viable embryos and a specificity of 60.5% for non-viable embryos in three independent datasets from different clinics. It demonstrated better predictive ability for embryo viability assessment compared to traditional embryologist methods. The increased accuracy of the Life Whisperer AI model could improve success rates in IVF (43).

A study by Huang analyzed more than 33,700 embryos using time-lapse images, developing a model of deep learning that predicts live birth with an average AUC of 0.968 in cross-validation and 0.957 in independent testing (44). Similarly, the new model named IVY used time-lapse videos from eight clinics to predict fetal heartbeat pregnancy, achieving an AUC of ~0.93, reproducible across laboratories (45).

A fundamental component of IVF is the evaluation and selection of viable embryos for transfer. Berntsen assessed the performance of a DL-based embryo selection model using time-lapse image sequences. The fully automated model was trained and evaluated on a large dataset from 18 IVF centers. It demonstrated performance at least equal to that of a state-of-the-art manual selection model while eliminating biases due to inter- and intraobserver variability (46).

Simpler but clinically useful models, such as nomograms to predict pregnancy failure in patients with poor ovarian response, use traditional predictive factors [age, basal follicle-stimulating hormone (FSH), number of high-quality embryos] and apply logistic regression (47).

Also, there are models that predict fertilization failure in conventional IVF cycles based on semen parameters, mature oocytes, and sperm morphology, among others (48).

The most important variables in prediction are maternal age, embryo quality, hormone levels on the day of human chorionic gonadotropin (hCG) administration, and the stimulation protocol. Feature selection using genetic algorithms can optimize model performance.

While AUC and accuracy are the most frequently reported performance metrics, these statistical measures only reflect the model’s discriminative ability and not its clinical utility. A truly useful predictive model should demonstrate measurable benefits in patient outcomes such as improved live birth rates, reduced time to sperm retrieval in mTESE, better treatment selection, or enhanced cost-effectiveness. Therefore, beyond internal validation, future research should prioritize prospective, multicenter implementation studies and randomized trials that assess these clinically meaningful outcomes.

Models such as FORTUNE provide a strong methodological example of internal and external validation, but broader, outcome-linked, multicenter studies remain essential to establish generalizability and real-world benefit. Authors of future predictive AI studies are encouraged to report both discrimination metrics and clinical impact indicators, ensuring that performance evaluation extends beyond statistical accuracy.

Thus, these types of AI models facilitate clinical decision-making and patient counseling on IVF success rates. However, their broader clinical adoption requires prospective validation, harmonized reporting standards, and adaptation to local contexts to ensure fair and generalizable use (49).

A summary of the evidence in this domain is provided in **Table 4**.

**Table 4:** A summary of evidence on AI-based predictive models.

Article	Clinical task	Data type	Model class	Dataset	Key metric	Validation level	Limitations	Evidence level
S Deghan et al. (39)	IVF success prediction (clinical pregnancy)	Clinical and laboratory variables	Random forest, ANN, SVM, AdaBoost	812 patients, single center	Accuracy of 89.8%	Internal validation	Single-center dataset, no external reproducibility and retrospective study	Low
E Seli et al. (40)	Prognosis grouping for euploid yield	Clinical predictors	Neural networks	10,774 cycles	AUC 0.83–0.86	Internal cross-validation	Single network and lacks lab-specific factors	High
R Bai et al. (41)	Pregnancy and live birth prediction	Clinical data	XGBoost, LightGBM	2,625 cycles, single center	AUC 0.999 (pregnancy), 0.913 (live birth	Internal validation	Possible overfitting, single center, and no external validation	Low–moderate
M Salih et al. (42)	Pregnancy or live birth prediction	Clinical and static embryo images	MLP, CNN	1,503 cycles	AUC 0.91	Hold-out and cross-validation	Small dataset and heterogeneity in clinical data	Moderate
M Milyea et al. (43)	Embryo viability	Static blastocyst images	CNN	8,886 embryos	Sensitivity 70.1%, Specificity 60.5%	External (multiclinic)	Limited to day-5 images and retrospective data	High
B Huang et al. (44)	Live birth prediction	Time-lapse videos	Deep CNN	>33,700 embryos, single center	AUC 0.968	Internal cross-validation and external test	Single center, retrospective and equipment-dependent	Moderate
D Tran et al. (45)	Pregnancy prediction	Time-lapse videos	DL	10,638 embryos	AUC 0.93	Internal cross-validation and external multiclinic	Endpoint limited to day-5	High
J Berntsen et al. (46)	Embryo selection	Time-lapse image sequences	CNN	115,832 embryos	AUC 0.67	External hold-out validation	Retrospective and varying protocols across clinics	High
F Li et al. (47)	Pregnancy failure risk	Clinical variables	Logistic regression	281 patients, single center	AUC 0.786	Internal validation	Small sample and single center	Low
L Xignan et al. (48)	Fertilization failure risk	Clinical and semen parameters	Logistic regression (multivariate)	1770 patients, single center	AUC: 0.756–0.776	Internal validation	Single center, no external validation, and retrospective study	Moderate
R AlSaad et al. (49)	Ovarian stimulation response prediction	Mixed: clinical, hormonal, imaging	ML and SVM	30 studies (mostly single center)	–	Internal validation	Heterogeneity in methods, mostly single-center studies, and no pooled metrics	Low

## The advantages of AI in the management of male infertility

AI has demonstrated remarkable potential in medicine through its ability to process vast amounts of data. AI algorithms can support embryologists by enabling faster, more standardized, and more reliable sperm selection, thereby improving embryo development and pregnancy outcomes (14).

### Improved diagnostic accuracy and reduction of subjectivity

AI is emerging as a powerful tool for enhancing sperm selection. Methods based on AI could improve the evaluation of some parameters such as motility and morphology. Motility is essential for sperm to reach the oocyte, but it has traditionally been difficult to assess due to the dynamic nature of movement. While early methods relied on fixing the sperm head to analyze tail beating, advances in imaging and software like SpermQ and Flagellar Analysis and Sperm Tracking (FAST) now allow detailed analyses of sperm motility (30).

Beyond motility, AI also offers the ability to examine sperm morphology (30). As previously discussed, DL algorithms can detect morphological abnormalities in unstained sperm cell images with an accuracy of 80.7% for acrosome defects (15).

Morphology is related to DNA integrity and is an important predictor of reproductive success. A CNN algorithm was developed that showed a correlation between actual and predicted DFI values based solely on sperm images. This could potentially serve as the basis for predicting other sperm morphology characteristics (30).

Integrating sperm selection technologies is expected to improve laboratory efficiency and increase the success of ART (30).

### Automation and diagnostic efficiency

AI models have also recently been used to help identify the rare sperm present within these testicular tissue samples. Currently, there are no automated, computer-assisted systems for identifying sperm in testicular tissue. Rather, it requires a clinician to spend hours manually searching for and retrieving the few sperm cells required for ICSI. This manual, tedious, and time-consuming task is inherently limited by human cognition and patience, leading to a significant risk of missing sperm. Aside from a lack of sperm production, an inability to find very rare sperm amid the millions of other cells in the specimen is one of the main reasons for mTESE failure. To ease this process and enhance testicular sperm retrieval rates, researchers have started exploring AI-based technologies for analyzing testicular tissue samples (15).

Wu et al. described the first CASA-like system that could be applied to testicular tissue samples (50). Overall, the CNN had a substantially lower precision than the embryologists (74% vs. 93%, respectively). This difference was attributed to the fact that the CNN had trouble identifying abnormal sperm, particularly those with bent tails or morphologic deformities and those obscured by microscopic artifacts. However, although it is not as accurate as an embryologist, the CNN was significantly more efficient. In fact, predictions were made on all 702 images in just 25 s, as opposed to the 2–3 h required by an embryologist (15).

In a more recent study, Lee et al. similarly developed a deep CNN to identify sperm cells within testicular tissue samples (51). This CNN was initially trained using samples of testicular tissue doped with a high density of fluorescently stained donor sperm (3,000–6,000 sperm cells per 30,000–50,000 testicular tissue cells). However, when tested on images from samples containing only a few sperm cells (10–200 sperm cells per 30,000–50,000 testicular tissue cells), the CNN achieved a similar positive predictive value (84.4%) and an even higher sensitivity (86.1%).

Though further studies are needed to externally validate and improve the accuracy of these models, automated sperm identification could play a role in evaluating samples obtained from mTESE procedures in the coming years. This technology is expected to be especially useful in low-volume centers, which may have limited technician availability and/or experience (15).

### Optimal sperm selection for assisted reproductive technologies

Although IVF is one of the most widely used treatments for infertility, its success rate is only approximately 45%, and outcomes can vary considerably across different centers (15). In the IVF/ICSI process, two steps remain particularly vulnerable to human subjectivity and variability: the selection of sperm and the selection of embryos. AI has the potential to standardize these procedures and reduce the subjectivity inherent in manual assessments. The latest studies on the use of AI in sperm selection have already been discussed. However, AI is also emerging as one of the most promising and objective approaches for embryo selection and predicting pregnancy outcomes (52). Several recent studies have applied AI-based models to predict embryo implantation (52). For instance, Tran et al. developed a model that can predict the likelihood of achieving a fetal heartbeat (45).

On the other hand, DL can also anticipate blastocyst quality based on static or time-lapse embryo images with high accuracy for each patient, as well as a CNN that could be prepared to identify specific regions in the embryo, which are analyzed by an algorithm to evaluate embryo quality. As it has been suggested, optimizing embryo selection could decrease the odds of multiple pregnancies and their associated risks (53).

### Prediction of clinical outcomes

#### Who should undergo varicocelectomy?

Varicocele, which is defined as an abnormal dilation of the pampiniform plexus of the spermatic cord, is detected in approximately 8%–15% of the general male population. However, it occurs more frequently in men with fertility issues, with up to 40% of those experiencing primary infertility and nearly 80% of those experiencing secondary infertility presenting varicocele. Its presence has been associated with altered semen parameters and hormone levels, as well as reduced pregnancy rates. Varicocele is one of the most common surgically correctable causes of male infertility. Nevertheless, the effectiveness of varicocelectomy in improving fertility outcomes remains debated. Current guidelines do not provide clear criteria for predicting which patients will achieve significant improvements after surgery (54). AI-based predictive models may offer a valuable tool for selecting men most likely to benefit from intervention (15).

Ory et al. used an ML algorithm to predict which patients may benefit from varicocele repair (55). This study collected data from 240 patients in two different cohorts: one was used to develop the algorithm, and the other was used to validate it. The authors demonstrated that a combination of FSH values, sperm concentration, and bilaterality could be used to create an algorithm to predict which varicocele patients would benefit from the intervention (56). This algorithm presented a sensitivity of 86.7%. However, the nomogram performed poorly in multicenter validation.

On the other hand, another random forest ML model was created. This model was developed using data from the University of Miami, and then it was externally validated using data from the University of Toronto. A total of 240 men were evaluated. The study offered high predictive accuracy (AUC 0.72). Although this model has not yet been validated by other centers, it provides a promising step for bringing AI into the clinical management of male infertility due to varicocele (15).

In parallel, advances in robotic-assisted microsurgery are transforming the operative management of varicocele. As highlighted by Napolitano et al., robotic platforms offer enhanced precision, tremor elimination, 3D visualization, and improved surgeon ergonomics, all of which contribute to safer and more effective ligation of spermatic veins (57). The integration of AI-driven decision support and intraoperative vision tools has the potential to complement robotic systems, optimizing both preoperative candidate selection and intraoperative navigation. This emerging synergy between robotics and artificial intelligence could pave the way toward truly personalized, image-guided microsurgical varicocelectomy in male infertility.

#### Which azoospermic men are most likely to have sperm present?

Obstructive azoospermia (OA) can easily be differentiated from non-obstructive azoospermia (NOA) based on clinical and laboratory parameters. However, which men with NOA could be benefit from a surgical sperm retrieval procedure remains difficult to distinguish. Predicting sperm retrieval rates (SSRs) in men with NOA has been challenging in recent years (15).

As a result, researchers have started to develop predictive models based on AI to overcome this clinical challenge. In particular, ML and DL approaches have been applied to estimate the likelihood of retrieving sperm during testicular biopsy (TESE) or mTESE (38).

Zeadna et al. used a type of ML model to predict the presence of sperm during TESE. A retrospective cohort of 119 NOA patients was examined to develop a predictive gradient-boosting tree model. Clinical variables were considered. Overall, the model was able to predict the presence of sperm correctly with an accuracy of 77.3% and an AUC of 0.81 (58). However, this study had several significant limitations, including its small sample size, retrospective nature, and lack of external validation. It also only included conventional TESE patients, not those undergoing mTESE (the gold-standard sperm retrieval technique for NOA patients), which may underestimate SSR (38). Nonetheless, these findings show that ML could be a valuable tool for predicting SSR in men with NOA. As models are refined using larger datasets and undergo external validation across diverse populations, their accuracy and clinical relevance will likely improve. Future research should aim to overcome these current limitations (15).

### Integration of complex data for personalized medicine

Advances in genomics have already led reproductive medicine and fertility treatment toward a precision medicine approach by AI (59).

Carrier genetic screening enables parents to choose embryos without specific mutations based on preimplantation genetic testing. In older patients undergoing IVF, preimplantation genetic testing could identify aneuploidy and select embryos with the greatest likelihood of resulting in a successful pregnancy. Otherwise, post-implantation genetic testing could also be used to diagnose trisomy 21 and other genetic abnormalities (59).

Integrating genomic data could have the potential to transform outcome prediction by identifying genetic markers associated with sperm quality or IVF success; for instance, whole-genome sequencing could be used to detect mutations that impair spermatogenesis (59).

Future work should focus on combining genomics with real-time analysis and hybrid AI–expert systems to further enhance IVF outcomes and extend the clear advantages demonstrated over conventional methods (59).

### The disadvantages of AI in the management of male infertility

AI will be a valuable tool in the next few years. However, it is still in its early stages, and its use in diagnostic and therapeutic settings is challenged by ethical, legal, and technological issues that need to be resolved. Nevertheless, AI has a lot of potential benefits in medicine, including in the fertility field. Due to the growing significance of technology, very promising results have been achieved (60).

However, examining biases and ethical implications prior to widespread application is crucial. This must be analyzed with every disruptive invention, particularly when the technology directly impacts patient outcomes and healthcare decisions. Even though AI is frequently presented as an impartial instrument, ethical issues remain (60).

### Lack of robust clinical validation and data quality evaluation

AI algorithms are only as good as the data on which they are based. But in the field of fertility, datasets are usually small, and AI algorithms are not specifically designed for fertility clinics (19).

While some previous studies have cited the need for large datasets to train the system as a limitation of AI, there is now a movement to create better resources and encourage collaboration between research centers in order to build adequate datasets that can power appropriate training (30).

At the same time, the reviewed studies used a variety of AI techniques, data sources, and applications within the field of male infertility. This heterogeneity makes it challenging to draw definitive conclusions and compare the performance of different AI models (16).

In addition, there may also be limitations regarding generalizability due to difficulties in standardizing ML methods. Variation in patient demographics, clinical practices, and laboratory procedures may introduce bias into the data (19).

For this reason, when an AI model is based on training in one clinic, it should be validated in independent cohorts (19). Replication across cohorts and prospective validation are also critical for identifying potential biases, evident when performance worsens in replication cohorts compared to training cohorts (53).

Furthermore, models should not be limited by strict inclusion criteria. Ideally, datasets should contain data from different clinics, with the testing data coming from a site that is different from those used for training and validation (19).

Nevertheless, the relatively small number of studies limits the ability to generalize the findings. Many of the reviewed studies are limited by small sample sizes and retrospective data collection, which may introduce selection biases and limit external validity. Most AI models have been trained on specific populations, raising concerns about their applicability to diverse patient demographics (16).

Therefore, to improve reliability, future research should prioritize large-scale, prospective studies with diverse datasets to ensure that AI models are robust and widely applicable (16). Large open datasets and methods developed specifically for use in the context of ART could lead to better results and understanding (19).

### Algorithmic bias, overfitting, and spurious correlations

Health datasets, on which AI is based, have also been shown to be profoundly biased (53). Due to the inherent bias and variability present in AI models, it is crucial to validate AI software thoroughly before using it for clinical decision-making or assistance. The collapse of an algorithm is a real concern for new systems that have been trained using poorly optimized datasets. Furthermore, it is unclear whether AI-based technology will be a valuable support to urologists, embryologists, and patients, or whether it could be misused as an inappropriate substitute for human expertise.

Despite the perception of objectivity, it is crucial to recognize that AI technology is not exempt from bias. AI models are constrained by the proficiency of their producers and the training dataset selected. Human biases can be introduced when selecting variables for an AI model, which can result in significant variables being excluded and confounding variables being included (60). In addition, bias can permeate every stage of AI program development and perpetuate systemic discrimination. For instance, the quality of imaging inputs may be limited by the imaging platform, image annotations, or resolution. Similarly, meticulous documentation of clinical outcomes is required to create an adequate training set, which can be challenging in certain scenarios, such as with untransferred cryopreserved blastocysts (60).

The most common bias is the limited ethnic diversity of the cohorts from which these datasets originate (53, 61). Additionally, the high financial cost of IVF limits access, leading to a known socioeconomic and racial bias (60). Moreover, non-diverse datasets can introduce biases, resulting in misleading predictions for different demographic groups (16). Consequently, such limitations can result in precision medicine that is more effective for certain populations than others and can also lead to misdiagnoses in underrepresented groups. This can potentially cause harm and exacerbate health disparities (53, 62).

This issue is often ignored because it challenges the widely held belief that technology, including AI, is an impartial and equitable system that does not perpetuate the defects of society. Another area in which AI-based algorithms can initially fall short is the inherent bias or variance in the training data. AI bias and variance affect the accuracy and stability of the algorithm, so the objective is to balance them as effectively as possible. A biased algorithm oversimplifies the connection between input and output data, leading to underfitting and making it unsuitable for handling datasets with numerous subtle relationships between data points. By contrast, excessive variance denotes the sensibility of an algorithm to detect small changes in the training data. This can cause overfitting, resulting in increased specialization at the expense of generalizability, as well as inaccurate prediction of outcomes from new, unseen input data. The ideal algorithm seeks an appropriate balance between complexity and flexibility, neither oversimplifying relationships nor ignoring legitimate patterns in the data (60).

Validating a new dataset and addressing the risk of erratic predictions or algorithm collapse remain significant challenges in the current literature. Moreover, data privacy and sharing concerns make external validation of any AI algorithm challenging, and caution must be exercised when evaluating any new AI system (60). One potential approach is to pool multiple datasets from various centers and sources to mitigate the influence of local bias and improve robustness (53).

Thus, it is crucial for clinicians to ensure that the algorithms used in their practice are as unbiased as possible in order to prevent exacerbating existing healthcare inequalities (60).

### Lack of standardization and regulatory oversight

Several obstacles have been identified in the application of AI in medicine. For instance, both AI and precision medicine are relatively unstandardized (53). Clinical outcomes must be carefully recorded to generate sufficient training data. This can be challenging in certain situations. For instance, when cryopreserved blastocysts are not transferred, little data are available for blastocysts that are subjectively morphology-selected (60).

In addition to data quality, external validation remains a major barrier. Data privacy and sharing issues impede the verification of AI algorithms among different cohorts. A thorough assessment is imperative before clinical acceptance. Therefore, to make sure that AI systems help provide ethical and patient-centered care, the highest standards of rigor must be maintained (60).

Regulatory frameworks also pose important challenges. While organizations like the Food and Drug Administration (FDA) and European Medicines Agency (EMA) enforce strict safety and accuracy requirements, their lengthy approval processes can be costly and time-consuming, particularly for smaller clinics in disadvantaged areas. Although regulation is essential for patient safety, streamlined pathways may help balance timely access to innovation with appropriate oversight (16).

Beyond regulation, assessing AI performance presents technical difficulties. Many different measures, including accuracy, precision, and/or sensitivity, are used to evaluate modern AI algorithms. This emphasizes the need to provide common training and testing criteria through objective, proven, current benchmarking datasets and instructions (15).

Proper evaluation and testing of AI systems in relation to outcomes and regulations is critical, as is a better understanding of the technical aspects and determining the practical value of AI models in the clinic (19). Moreover, standardizing the use of AI in ART is essential for achieving more transparent, comparable, and reproducible results.

### Limited transparency and explainability

A related problem is that the representativeness and size of training datasets are limited in domains where patient data are used. Although these datasets are typically small, some patient-derived ones, like the Human Sperm Head Morphology dataset (HuSHeM), are made publicly available. However, due to privacy protections like the Health Insurance Portability and Accountability Act (HIPAA) regulations, the intricacy of legacy electronic health records and the competition among medical centers make it difficult to share data to create a large and diverse database for AI training (53). In the future, new ML techniques such as federated learning may be able to overcome some of these data-sharing problems (63).

### Ethical, privacy, and legal considerations

Concerns about data privacy and sharing make it challenging to validate any AI algorithm externally. Moreover, caution must be exercised when evaluating any new AI system (60). Due to data privacy and ethical considerations, patient data and treatment information are not easily obtained for research purposes. As a result, the amount of patient-related data available to train the AI model is limited. This presents difficulties for DL methods, which mostly rely on image and video categorization and require a large volume of diverse data in order to be generalizable (19).

Beyond these limitations, data privacy and ownership present another challenge. AI algorithms process highly private reproductive data, including sperm analysis, medical histories, and genetic profiles. Without robust security measures such as encryption, this information could be vulnerable to breaches or commercial exploitation by clinics and technology businesses. Data ownership requires well-defined legal systems to guarantee informed permission and safeguard patient autonomy. Likewise, anonymization should be mandatory before data are shared with other institutions. Transparent AI systems and legal guidelines are essential for defining liability. Future research and policy development must focus on these issues to ensure the ethical implementation of AI in healthcare (16).

In order to ensure the safe and ethical deployment of AI in healthcare, it is essential that both developers and healthcare professionals must be aware of the impact of bias and variance within algorithms. Biases can be introduced at various stages of algorithm development and perpetuate socioeconomic, demographic, and racial inequalities in healthcare delivery (64). A lack of diversity in training data can result in biased algorithms that fail to consider the nuances of patient care, ultimately impacting patient outcomes. Therefore, AI algorithms must be developed with appropriate care and rigor to ensure that they meet ethical standards and promote patient-centered care (60).

Clinicians must also ensure that the algorithms they use in their practice are as unbiased as possible in order to prevent exacerbating existing healthcare inequalities (60). While portable AI solutions may offer some relief in areas with limited resources, comprehensive global policies are essential to ensure fair distribution (16). An accurate training dataset that reflects clinical situations and is sufficiently varied to be generalizable to several patient populations must be created (60). Dealing with moral issues, such as accessibility, misuse, psychological consequences, data security, and accountability, will assist in guaranteeing that AI-driven reproductive healthcare remains fair and ethical (16).

In these circumstances, patients must be informed of and agree to the dissemination of their information. To guarantee patient privacy and prevent any mismanagement of personal information, data should be anonymized before distribution to another institution. Alongside these fundamental measures, the international community should consider modernizing existing privacy legislation and rules to reflect the increasing application of AI models in modern medicine (15).

In this context, the recently approved EU Artificial Intelligence Act highlights the importance of making use of AI in healthcare more responsibly and in ways that promote trustworthiness (65). The regulation acknowledges that medical AI systems can pose serious ethical and safety challenges, and for that reason, it calls for transparency, clear accountability, and active human oversight at every stage (from design to real-world use). The Act also emphasizes the need to understand where data come from and to maintain high standards of quality, as well as to continuously evaluate the performance of these systems. It seeks to protect the rights of patients, safeguard sensitive information, and foster genuine trust in the use of AI within clinical practice.

### Displacement of clinical judgment

The implementation of AI in existing algorithms is interrupted by the requirement for AI software to connect with current, clinically validated workflows. As most AI algorithms are based on pattern matching and do not imply any reasoning, therefore, these algorithms cannot manage borderline cases as well as a medically trained human. Integrating AI and human–machine collaborations is one approach that is currently being researched in this field. Such collaborations between humans and AI could become commonplace in the future. For instance, AI could potentially make largely automated decisions in most cases, but some would require complex reasoning that AI cannot yet perform (53).

## Conclusion

The integration of AI into semen analysis represents a significant advance for the standardization of diagnostic methods in the assessment of male fertility, making it more objective. The automation of the semen-analysis parameters such as morphology, motility, and sperm concentration has reduced the subjectivity inherent in manual analysis, increasing diagnostic accuracy and the reproducibility of results. AI learning models such as SVM, CNN, and R-CNN have demonstrated the ability to classify sperm with higher levels of accuracy than traditional methods.

Moreover, evidence suggests that AI models (especially DL applied to time-lapse imaging and validated commercial tools) improve the ability to stratify embryos and estimate pregnancy probability/fetal heartbeat compared with purely human assessments.

However, AI still has certain limitations. Most studies remain retrospective or lack robust validation. They use small and heterogeneous datasets, which makes it difficult to generalize or validate the results. Furthermore, the absence of standardized protocols and the lack of a direct correlation between parameters and clinical results of ART restrict their implementation in clinical practice. Multicenter prospective trials demonstrating real impact on live births and clinical safety are lacking.

Despite all this, the integration of multimodal approaches combining clinical information, microscopic images, and DNA data shows that AI will contribute to more accurate diagnoses and predictions in the coming years. It would enable better sperm selection and personalized treatment. However, large-scale validation studies, standardized databases, and the creation of transparent and reproducible algorithms are still needed.

Looking forward, meaningful progress in this field will depend on a coordinated research and implementation agenda centered around five priorities. Firstly, large-scale, multicenter and demographically diverse datasets must be developed to overcome current bias and enhance generalizability. This may lead to better model calibration, improved external validation AUCs, and greater population-level consistency. Secondly, the creation of standardized data collection is essential to ensure comparability and reproducibility across laboratories. Progress could be assessed by the proportion of studies adhering to unified protocols. Thirdly, multimodal AI architectures are needed to integrate clinical variables, microscopy images, time-lapse data, and molecular or genomic markers. These adjustments will improve the quality of diagnosis and treatment or even improve the embryo-selection efficiency. Fourth, prospective clinical studies must demonstrate measurable improvements in outcomes; for instance, success should be defined by higher live-birth rates per cycle or shorter time-to-pregnancy. Finally, the development of transparent, reproducible, and clinically interpretable AI systems should guide future implementation efforts, with feasibility assessed through clinician-adoption rates, interpretability scores, and reductions in algorithmic bias.

By clearly defining and systematically addressing these five priorities, the field can move beyond preliminary studies and advance toward AI systems that are clinically reliable, safe, and applicable across different settings. Developing systems that improve diagnostic accuracy and embryo and sperm selection and support personalized treatment decisions will increase the likelihood of successful assisted reproduction. This roadmap transforms the current evidence from a descriptive overview into a practical foundation for future research and clinical translation.
